# Silencing of CD44 Gene Expression in Human 143-B Osteosarcoma Cells Promotes Metastasis of Intratibial Tumors in SCID Mice

**DOI:** 10.1371/journal.pone.0060329

**Published:** 2013-04-02

**Authors:** Ana Gvozdenovic, Matthias J. E. Arlt, Carmen Campanile, Patrick Brennecke, Knut Husmann, Walter Born, Roman Muff, Bruno Fuchs

**Affiliations:** Laboratory for Orthopedic Research, Department of Orthopedics, Balgrist University Hospital, Zurich, Switzerland; University of Patras, Greece

## Abstract

Osteosarcoma (OS) is the most frequent primary malignant bone cancer in children and adolescents with a high propensity for lung metastasis. Therefore, it is of great importance to identify molecular markers leading to increased metastatic potential in order to devise more effective therapeutic strategies that suppress metastasis, the major cause of death in OS. CD44, the principal receptor for the extracellular matrix component hyaluronan (HA), is frequently found overexpressed in tumor cells and has been implicated in metastatic spread in various cancer types. Here, we investigated the effects of stable shRNA-mediated silencing of CD44 gene products on *in vitro* and *in vivo* metastatic properties of the highly metastatic human 143-B OS cell line. *In vitro*, CD44 knockdown resulted in a 73% decrease in the adhesion to HA, a 57% decrease in the migration rate in a trans-filter migration assay, and a 28% decrease in the cells' capacity for anchorage-independent growth in soft agar compared to the control cells, implicating that CD44 expression contributes to the metastatic activity of 143-B cells. However, making use of an orthotopic xenograft OS mouse model, we demonstrated that reduced CD44 expression facilitated primary tumor growth and formation of pulmonary metastases. The enhanced malignant phenotype was associated with decreased adhesion to HA and reduced expression of the tumor suppressor merlin *in vivo*. In conclusion, our study identified CD44 as a metastasis suppressor in this particular experimental OS model.

## Introduction

Osteosarcoma (OS) is the most common primary malignant bone cancer in children and adolescents, characterized by the presence of spindle-like tumor cells which produce immature bone or osteoid. The overall incidence is three patients/million/year with the median peak at the age of 16 [1]. OS has a high propensity for metastasis to the lung and bones. Twenty percent of the patients have detectable metastases already at the time of diagnosis and eighty percent of the patients who initially present with localized disease subsequently develop metastases [2], [3]. Significant clinical improvements over the past several decades through the use of combined chemotherapy and surgery have led to a dramatic increase in the survival of patients with localized disease. However, patients with metastatic or recurrent disease continue to have a very poor prognosis, with <20% long term survival [3]. Therefore, it is of great importance to elucidate the molecular mechanisms of OS metastasis. A detailed understanding of these mechanisms will in the future guide the design of novel treatment strategies and the development of corresponding metastasis suppressive compounds that will finally help to improve the survival of OS patients.

Previous studies revealed evidence for important functions of CD44 gene products during metastatic spread of numerous types of tumors [4] and respective proteins were therefore considered as targets for anti-metastatic treatment. Moreover, several reports have described prognostic power of CD44 gene products in different cancer types [5], [6], [7]. CD44 gene products are transmembrane proteoglycans, which act as cell-cell and cell-matrix adhesion molecules and principle receptors of hyaluronan (HA) [8]. They exist as multiple isoforms that are generated from a single primary gene transcript by alternative splicing combined with varying posttranslational glycosylation [9]. As a result, the extracellular domains of CD44 isoforms consist of variable regions, encoded by variant exons, and are glycosylated to different extents. The standard isoform CD44s, also described as CD44H (hematopoietic), is the smallest CD44 gene product and does not contain any of the variable extracellular regions encoded by the variant exons. The structural differences in the extracellular domains of the CD44 isoforms largely define a wide range of biological functions in development, inflammation, haematopoiesis, wound healing, immune response and cancer [4]. In tumor biology, evidence has accumulated that metastasis in different types of cancer is directly or inversely related to the expression of CD44 gene products, depending on the expression pattern of CD44 isoforms and on the organ of origin. Several studies have shown up-regulated expression of CD44 isoforms in many human tumors, including gastric cancer, pancreatic cancer, lung and renal cell cancer [6], [10], [11], [12]. Some reports point to important functions of CD44s in tumor progression [13]. However, in other tumor types, such as neuroblastoma and prostate cancer, the absence of CD44 gene products correlated with poor prognosis [7], [14]. In prostate carcinoma cells, CD44 was even considered as a metastasis-suppressor gene [4], [15]. Thus, the functions of CD44 gene products in tumor biology are controversially discussed.

Little is known on potential functions of CD44 gene products in OS pathophysiology and in OS metastasis in particular. Two immunohistochemical studies of OS tissue specimens revealed discrepant results. Kim et al. reported that overexpression of the CD44v5 isoform (CD44s with an additional amino acid sequence in the extracellular domain encoded by variant exon 5) in tumor tissue correlated significantly with metastatic disease and, consequently, lower survival rates of the patients [16]. In the tumor samples analyzed by Kuryu et al., on the other hand, a poor prognosis for OS patients correlated with overexpression of the CD44 isoforms that contain in the extracellular domain the amino acid sequence encoded by variant exon 6 (CD44v6) [17]. In both studies, immunostaining with CD44 antibodies detecting all expressed CD44 isoforms in tumor tissue together (pan CD44 antibodies) was not a prognostic indicator.

As outlined before, HA is the principle ligand of CD44 gene products. In OS, a few studies with established OS cell lines revealed evidence for a tumor promoting effect of HA. Hosono et al. showed that *in vitro* and *in vivo* tumorigenic properties of the OS cell lines MG63 and LM8 were inhibited by HA oligosaccharides that perturbed the HA-rich pericellular matrix [18]. A study performed by Nishida et al. showed diminished retention of HA and inhibition of tumorigenicity of MG63 OS cells upon antisense inhibition of HA synthase (HAS)-2 [19]. HAS-3-derived HA enhanced the cellular properties required for OS metastasis, such as proliferation, invasion and degradation of extracellular matrix *in vitro* [20].

The controversial discussions on the relevance of CD44 gene products in cancer biology in general and the limited knowledge on their biological functions in OS and metastasis in particular prompted us to perform the here reported CD44 silencing study in an intratibial OS xenograft model in SCID mice that makes use of the human highly metastatic 143-B cell line and reproduces the human disease with metastasis to the lung. The expression of CD44 gene products, predominantly CD44s, in the 143-B OS cell line, found to be representative for the CD44 isoform expression pattern in other OS cell lines, was stably downregulated by retroviral expression of shRNA. The effects of this manipulation in 143-B cells on the metastatic behavior *in vitro* and on intratibial tumor growth and lung metastasis in SCID mice were investigated.

## Materials and Methods

### Cell culture and transduction

Human 143-B OS cells (CRL-8303) were obtained from American Type Culture Collection (ATCC, Rockville, MD). The cells were cultured in DMEM (4.5 g/l glucose)/HamF12 (1∶1) medium (Invitrogen; Carlsbad, CA), supplemented with 10% heat-inactivated FCS (GIBCO, Basel, Switzerland), at 37°C in a humidified atmosphere of 5% CO_2_/95% air. CD44 expression was stably silenced by retroviral expression of shRNA in 143-B cells that were transduced with a *LacZ* gene for tumor cell identification by X-gal staining in mouse tissues [21]. Retroviral constructs in the pSirenRetroQ vector (Clontech; Paolo Alto, CA) coding for CD44 transcript-targeting shRNA (shCD44) and for non-targeting control shRNA (Ctrl shRNA) were kindly provided by Prof. Ivan Stamenkovic (Lausanne, Switzerland) [22]. Retroviral particles containing shCD44 or Ctrl shRNA constructs or the empty pSirenRetroQ vector (EV) with a puromycin resistance gene were produced in HEK293-T cells according to a protocol reported by Arlt et al. [23]. *LacZ*-transduced 143-B cells were infected with retroviruses by incubation for 48 h in virus-containing medium supplemented with 8 µg/ml polybrene. Retrovirus infected cells were subsequently selected and maintained in cell culture medium containing 2 µg/ml puromycin (Invitrogen). The selection revealed *LacZ-*expressing 143-B EV, 143-B shCD44 and 143-B Ctrl shRNA sublines. Prior to animal experiments, 143-B shCD44 cells were further enriched by incubation in tissue culture medium on HA-coated plates (100 µg/cm^2^; Sigma Aldrich, St. Luis, MO) at 37°C for 10 min that removed cells with inefficiently silenced CD44 expression. Non-adherent 143-B shCD44 cells in the supernatant were collected and used for animal experiments.

### Immunocytochemistry

Cells were allowed to grow to subconfluence on glass microscope cover slips in 24-well plates. After washing with PBS, the cells were fixed with 4% formalin in PBS at room temperature (RT) for 20 min. Permeabilization and non-specific antibody binding to cell monolayers were achieved by preincubation at RT for 30 min in DMEM/F12 (1∶1) medium containing 0.1% BSA and 0.1% saponin (blocking medium). The cells were then incubated at RT for 2 h with the primary pan CD44 antibody (Hermes3, kindly provided by Dr. Sirpa Jalkanen, Turku, Finland; 2 µg/ml in blocking medium). After extensive washing with blocking medium, secondary Alexa Fluor 546-labeled antibodies to mouse IgG (Invitrogen) at 1∶200 final dilution were added and the cells incubated in the dark for 30 min. F-actin was stained with Alexa Fluor 488–labeled phalloidin (Invitrogen) and cell nuclei were visualized with DAPI. The coverslips were then washed with PBS and dipped in H_2_O and then mounted in Immomount (ThermoScientific; Waltham, MA). Fluorescence was examined with a Nikon Eclipse E600 microscope equipped with appropriate filter blocks (Nikon Corporation, Tokyo, Japan).

### Western blot analysis

Cells were lysed by incubation at 4°C for 1 h on a moving carrousel in lysis buffer consisting of 50 mM Tris/HCl (pH 7.5), 150 mM NaCl, 1% NP40, 0.5% deoxycholic acid, 0.1% sodium dodecyl sulfate (SDS), 1 mM dithiothreitol (DTT), 1 mM phenylmethylsulphonyl fluoride (PMSF) and 10 mg/ml aprotinin. Cellular debris were pelleted by centrifugation at 16060×g and 4°C for 20 min. Equal amounts of proteins in supernatants were separated by 8% SDS-PAGE and then blotted onto Hybond-ECL membranes (GE Healthcare, UK). CD44 and β-actin were detected with mouse monoclonal Hermes3 antibodies (1 µg/ml) and antibodies to β-actin (Chemicon, Dietikon, Switzerland, final dilution 1∶10000), respectively. HRP-conjugated secondary antibodies were purchased from Santa Cruz Biotechnologies. Peroxidase activity was visualized with Immobilon chemoluminescence substrate (Millipore, Billerica, MA) and a VersaDoc™Imaging System (Bio-Rad; Hercules, CA).

### Adhesion assay

Adhesion assays were performed in 96-well plates. The wells were coated with 100 µl per well of 1 mg/ml high molecular weight HA diluted in PBS (333 µg/cm^2^ of HMW-HA; Sigma-Aldrich) at 4°C over night. They were then washed with PBS and blocked with heat-denatured (HD) 1% BSA in PBS at RT for 1 h. Non-coated wells or wells coated with HD-BSA alone were used as controls. Cells grown to subconfluence were detached with accutase (Sigma-Aldrich) and 10^4^ cells per well were seeded in triplicates and allowed to adhere at 37°C for 30 min. Non-adherent cells were removed by washing and adherent cells were then fixed with 10% formalin in PBS at RT for 15 min and subsequently stained with 0.05% crystal violet in H_2_O at RT for 15 min. Photos of an area of 3.6 mm^2^ were taken with an AxioCam MRm camera connected to the Zeiss Observer.Z1 inverted microscope adjusted to 4× magnification. The number of adherent cells was determined with ImageJ software. The percentage of adherent cells was calculated from the total number of adherent cells per well divided by the total number of seeded cells and multiplied by 100. The percentage of adherent 143-B Ctrl shRNA and 143-B shCD44 cells was normalized to the percentage of adherent 143B EV cells set to 100%. The data of three independent experiments are presented.

### Migration assay

Transwell migration assays were performed as recently reported [23]. Briefly, 5−10×10^3^ cells were allowed to migrate at 37°C for 6 h through 8 µm porous polycarbonate filters of cell culture inserts (Becton Dickinson, San Jose, CA) fitting into 24-well plates. Cells remaining on the upper side of the filter insert were considered as non-migrating cells and removed by wiping with a cotton swab. Migrated cells on the lower side of the filters were fixed with 10% formalin in PBS, permeabilized with 50 µM digitonin (Calbiochem; Switzerland) and stained with 300 nM Picogreen in PBS (Invitrogen) at RT for 15 min. Three randomly selected filter areas of 0.58 mm^2^ per insert (two filters per cell line) were photographed with an AxioCam MRm camera connected to the Zeiss Observer.Z1 inverted microscope equipped with an appropriate filter block for blue excitation and set to 10× magnification. The number of cells that migrated to the lower side of the polycarbonate filters was determined with ImageJ software. The results are presented as described for the adhesion assay. The experiments were carried out at least three times.

### Proliferation assay

Subconfluent, logarithmically growing cells were trypsinized and 5×10^4^ cells in 2.5 ml of cell culture medium were seeded in triplicates in 12.5 cm^2^ flasks and allowed to grow for between 1 and 5 days and collected at one-day intervals by trypsinization. The cell number/flask was determined by counting aliquots of harvested cells in a Neubauer chamber. The equation N = N_o_ e^kt^ was used to calculate the doubling time during logarithmic growth.

### Soft agar colony formation assay

Experiments were carried out in 6-well plates. A bottom agar layer in individual wells was generated with 1.5 ml of 0.5% DNA grade agarose (Promega, Madison, WI) in cell culture medium. The plates were kept at 4°C until use. 2×10^4^ cells in 1.5 ml of 0.35% agarose in cell culture medium were seeded per well in triplicates on top of the bottom agar layer. The cells were cultured at 37°C for 24 h before 2 ml per well of cell culture medium with penicillin/streptomycin/amphotericin B (PSA, 1∶100; Invitrogen) were added. The medium was replaced every 3 days and the cells were cultured for 14 days. Cell colonies were then stained with 2 ml 0.005% crystal violet at 4°C overnight. Three randomly selected areas per well were photographed with an AxioCam MRm camera connected to a Zeiss Observer.Z1 inverted microscope set to 4× magnification. ImageJ software was used to determine the number and the size distribution of colonies. The mean number of colonies per well formed by the individual manipulated cell lines was calculated and normalized to that of 143-B EV cells set to 100%. The experiments were repeated four times in triplicates.

### Intratibial xenograft OS mouse model

Eight week old SCID/CB17 immunocompromised mice were obtained from Charles River Laboratories (Sulzfeld, Germany) at least 14 days before the start of the experiment. The animal experiments were approved (License Number 129/2009) by the Ethics Committee of the Veterinary Office of the Kanton Zürich and were conducted in accordance with the guidelines of the “Schweizer Bundesamt für Veterinärwesen”. On day 0 of the experiments, 2×10^5^ of 143-B cells (engineered as described) in 10 µl of PBS/0.05% EDTA containing Matrigel (Becton-Dickinson; Franklin Lake, NJ) were injected intratibially. After the injections, the health condition of the mice was closely monitored. Tumor development was examined weekly by X-ray with an MX-20 DC Digital Radiography System (Faxitron X-Ray Corporation, Lincolnshire, IL) and by caliper measurements of the length and the width of the tumor leg from which the tumor volume was calculated with the formula V =  length × width^2^/2. The mice were sacrificed 21 days after tumor cell injection and in situ lung perfusion was performed as described [23]. Organs collected at sacrifice were fixed in 2% formaldehyde at RT for 30 min, washed three times with PBS and *LacZ* gene expressing tumor cells were stained with 5-bromo-4-chloro-3-indolyl-β-D-galactoside (X-Gal) staining solution at 37°C for at least 3 h as described [24], [25]. The indigo-blue stained metastases on the lung surface were counted under the microscope. The animal experiments were carried out three times. The data of a representative experiment are shown.

### Immunohistochemistry

Tumors and lungs previously fixed in 4% formaldehyde were dehydrated through serial incubation in 70%, 96%, 100% ethanol and xylene and then embedded in paraffin. Sections of 6 µm were mounted onto slides, deparaffinized and rehydrated and then heated in 0.1 M citrate buffer (pH 5.8) for antigen retrieval. Endogenous peroxidase was inactivated by incubation of the tissue sections in 3% H_2_O_2_ at RT for 10 min. Non-specific binding of antibodies to tissue sections was blocked by incubation at RT for 1 h in Tris buffered saline (TBS; 50 mM Tris, 150 mM NaCl, pH 7.4) that contained 10% goat serum (Vector Laboratories; Burlingame, CA) and 0.1% Tween (Sigma Aldrich). Primary Hermes3 CD44 antibodies (2 µg/ml in blocking solution), and antibodies to merlin NF2 (Santa Cruz Biotechnologies; 4 µg/ml) and to Ki67 (Abcam; Cambridge, UK; 4 µg/ml) were then applied and the slides incubated at RT for 1 h. After washing with TBS, the slides were incubated with secondary biotinylated goat anti-mouse IgG (Vector; 1∶200) at RT for 1 h. Slides incubated with secondary antibody alone served as negative controls. After another wash with TBS, the sections were incubated with avidin-conjugated peroxidase (ABC kit; Vector Laboratories) at RT in the dark for 30 min, washed again with TBS, and then incubated with the peroxidase substrate AEC (Dako; Glostrup, Denmark) for staining. Finally, the slides were briefly counterstained with hematoxylin. Recombinant mouse CD44 Fc chimera (R&D Systems, Minneapolis, MN; 10 µg/ml) were used for the staining of HA in tissue sections with the standard protocol for immunostaining excluding antigen retrieval. For negative controls, tissue sections were treated with hyaluronidase (200 U/ml; Sigma Aldrich) at 37°C overnight prior to HA staining, or the CD44 Fc chimera were preincubated with HA (1 mg/ml; Sigma Aldrich) before application to the slides.

### Statistical analysis

Differences between means were analyzed by the Student t-test and p<0.05 was considered significant. The results are presented as means ± SEM.

## Results

### shRNA-mediated silencing of the CD44 gene in the human metastatic 143-B OS cell line diminishes *in vitro* metastatic properties

**Figure 1:**
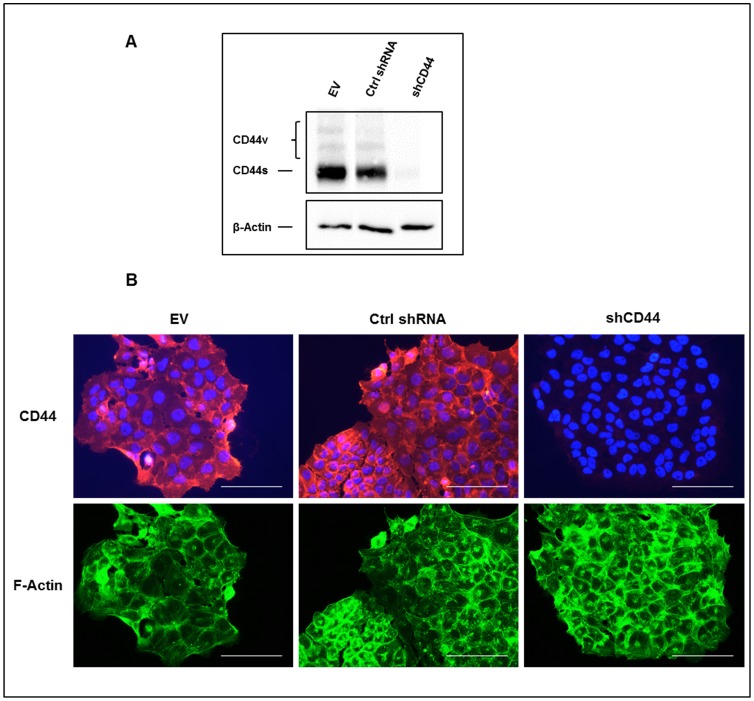
shRNA-mediated downregulation of CD44 expression in 143-B OS cells. (A) Western blot analysis with the panCD44 Hermes3 antibody of total CD44 gene-derived protein products in extracts of 143-B EV (EV), 143-B Ctrl shRNA (Ctrl shRNA) or 143-B shCD44 (shCD44) cells. β-Actin was used as a loading control. (B) Cell immunostaining of CD44 (red) in saponin permeabilized 143-B EV (EV), 143-B Ctrl shRNA (Ctrl shRNA) or 143-B shCD44 (shCD44) cells. Actin filaments (green) and cell nuclei (blue) were visualized with Alexa Fluor 488-labeled phalloidin and DAPI, respectively. Bars, 100 µm.

**Figure 2:**
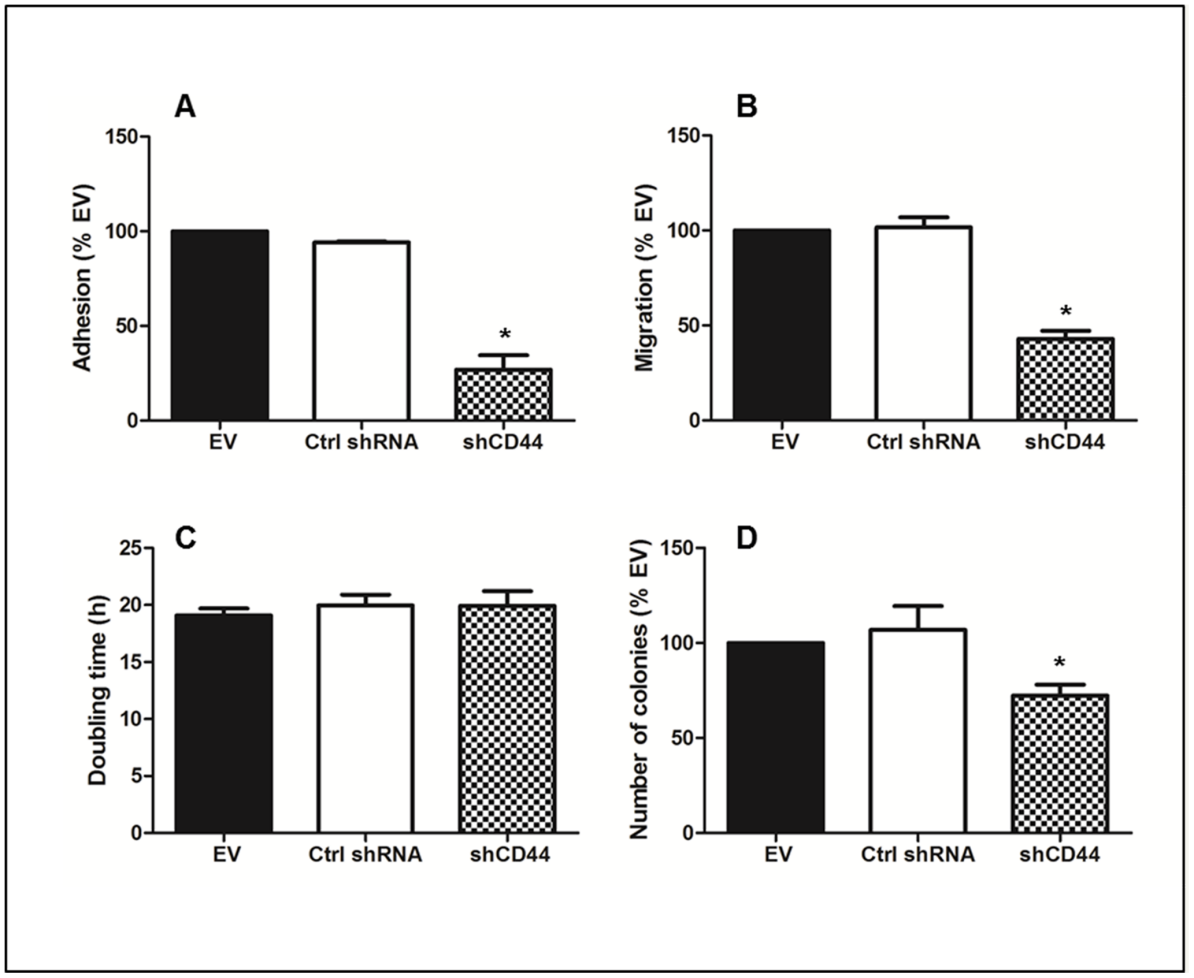
Effects of CD44 silencing on *in-vitro* malignant properties of 143-B OS cells. (A) Adhesion to HA (n = 3), (B) trans-filter migration (n = 6), (C) proliferation (n = 3) and (D) anchorage-independent growth (n = 4) of 143-B EV (EV), 143-B Ctrl shRNA (Ctrl shRNA) or 143-B shCD44 (shCD44) cells. Values represent the mean ± SEM; *, p<0.05.

An analysis in 143-B cells of the products derived from the CD44 gene revealed predominant expression of the standard CD44s isoform, a finding that was consistent with observations in other established as well as primary human OS cell lines (not shown). Based on the previously reported malignant phenotype of 143-B cells *in vivo*, which, upon intratibial injection, nicely reproduced the human disease with primary osteolytic bone lesion that metastasize to the lung [26], 143-B cells stably expressing a *LacZ* gene were used to study the biological relevance of CD44 molecules in OS aggressiveness. Retroviral transduction of 143-B cells with a vector for stable expression of CD44 gene transcript-targeting shRNA revealed effective downregulation of CD44 gene-derived protein products in cell extracts and in the cell monolayers visualized by immunocytochemistry (Figure 1A and B). This was not observed in 143-B cells transduced with empty-vector retroviruses or with viruses producing non-specific control shRNA. Staining of actin filaments, on the other hand, clearly demonstrated that morphological features of the three cell lines were not affected by the described manipulations. This silencing of the CD44 gene in 143-B cells reduced their capacity to adhere to HA by 73 ± 7.5% (p<0.02) compared to that observed with 143-B EV cells (Figure 2A). The adhesion of 143-B Ctrl shRNA cells with maintained CD44 expression, on the other hand, was indistinguishable from that of 143-B EV cells. Similarly, the CD44 silencing observed in 143-B shCD44 cells reduced the migration rate by 57 ± 4.2% (p<0.0001) compared to that of 143-B EV cells, which was also indistinguishable from that of 143-B Ctrl shRNA cells (Figure 2B). Interestingly, CD44 silencing had no effect on proliferation of 143-B cells in 2D culture (Figure 2C). Cell cycle distribution assessed by propidium iodide staining followed by flow cytometry was identical in the respective cell line populations (Figure S1). The number of 143-B shCD44 cell colonies growing anchorage-independent in soft agar, on the other hand, was 28 ± 6% (p<0.02) lower than that of 143-B EV cells, which was comparable to that of 143-B Ctrl shRNA cells (Figure 2D). The size of growing colonies of the three cell lines in soft agar did not differ (not shown).

CD44 silencing in 143-B OS cells enhances their malignancy in SCID mice

**Figure 3:**
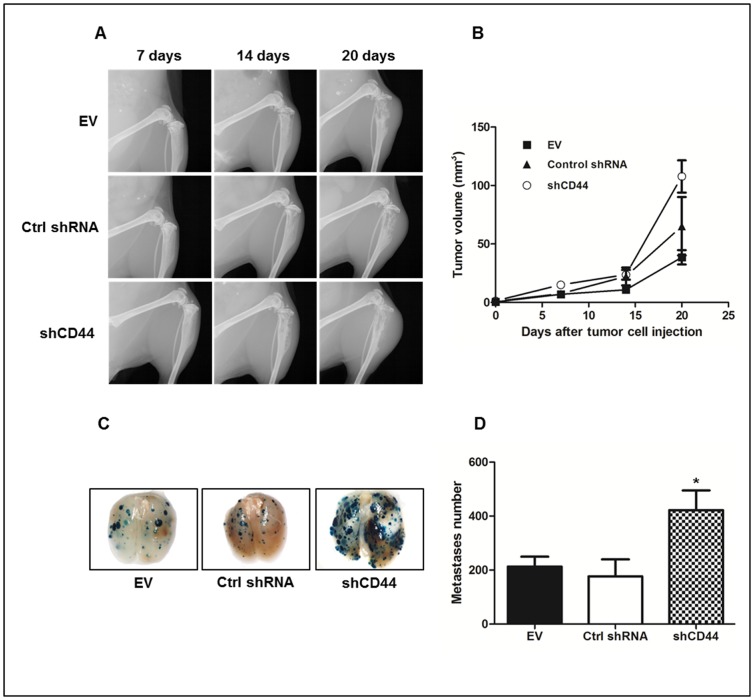
Effects of CD44 silencing on intratibial primary tumor growth and lung metastasis of 143-B OS cells in SCID mice. (A) Primary tumor development over time monitored by X-ray or (B) by tumor leg volume measurement at indicated time points in mice intratibially injected with 143-B EV (EV) (n = 9), 143-B Ctrl shRNA (Ctrl shRNA) (n = 6) or 143-B shCD44 (shCD44) (n = 9) cells. (C) Representative images and (D) quantification of X-gal stained (blue) metastases on whole-mounts of lungs collected from mice intratibially injected with 143-B EV (EV) (n = 9), 143-B Ctrl shRNA (Ctrl shRNA) (n = 6) or 143-B shCD44 (shCD44) (n = 9) cells. Values are expressed as mean ± SEM; *, p<0.05.

The results of the *in vitro* characterization of the malignant properties of 143-B shCD44, - Ctrl shRNA and - EV cells suggested that stable shRNA-mediated silencing of the CD44 gene in 143-B cells might also affect the development *in vivo* of intratibial 143-B cell-derived primary tumors and lung metastasis. Three groups of SCID mice were therefore intratibially injected with 143-B shCD44, - Ctrl shRNA or - EV cells, respectively. Fourteen days after tumor cell injection, similar osteolytic lesions, characteristic for 143-B cell-derived tumors, were recognized by X-ray in all three groups of mice (Figure 3A). Somehow unexpectedly, caliper measurements of the volume of the tumor legs over time revealed growth of intratibial primary tumors to a significantly (p<0.001) larger final size at sacrifice on experimental day 20 in mice injected with 143-B shCD44 cells (108 ± 14 mm^3^, n = 9) than in animals that received 143-B EV cells (39 ± 6 mm^3^, n = 9) (Figure 3B). The mean size of tumors developing in mice injected with 143-B Ctrl shRNA cells (65 ± 25 mm^3^, n = 6) was intermediate, but not significantly different from that in mice injected with 143-B shCD44 or with 143-B EV cells, most probably due to the fact that Ctrl shRNA group consisted of only 6 animals that exhibited greater heterogeneity in tumor size than the other experimental groups. Two of the 6 mice of 143-B Ctrl group, in particular, developed remarkably larger primary tumors than the other 4 mice of this group and all mice in the 143-B EV group that were similar in size. Interestingly, beside the described larger size of 143-B shCD44 compared to 143-B EV and 143-B Ctrl shRNA cell-derived primary tumors, the mean number of metastatic lesions on lung surfaces at sacrifice, detected by X-gal staining, were also found significantly increased 2-fold (p<0.05) and 2.4-fold (p<0.05) in mice with 143-B shCD44 cell-derived tumors compared the numbers counted on the lungs of animals injected with 143-B EV or 143-B Ctrl shRNA cells, respectively (Figure 3C, D).

**Figure 4:**
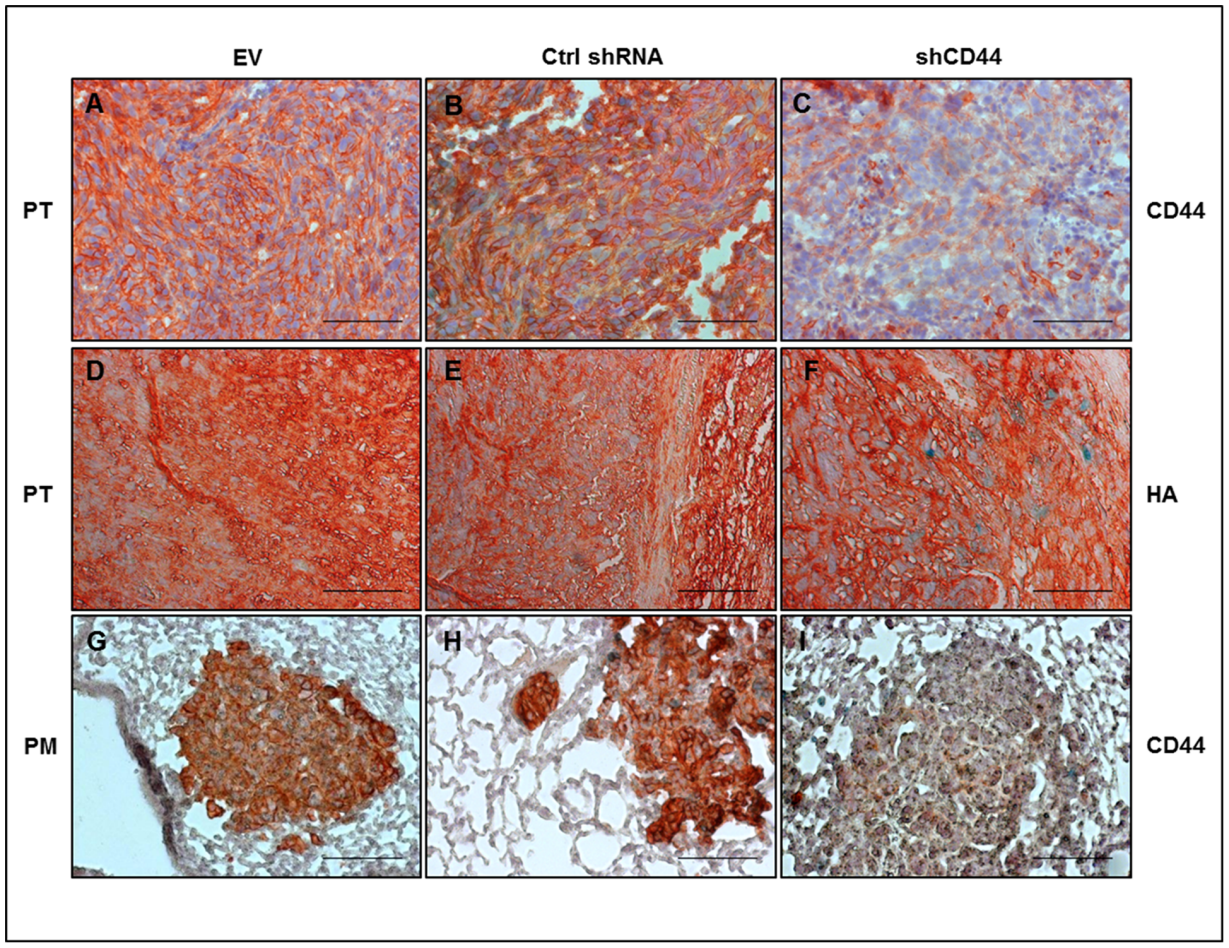
*Ex-vivo* immunohistochemical analysis of shRNA mediated CD44 silencing in 143-B-lacZ OS cell-derived intratibial primary tumors and pulmonary metastases. Robust expression of immunoreactive CD44 observed in primary tumor (PT) tissue and pulmonary metastases (PM) derived from 143-B EV (EV) (A,G) and 143-B Ctrl shRNA (Ctrl shRNA) cells (B,H) was found suppressed in tumor tissue (C) and lung metastases (I) derived from 143-B shCD44 (shCD44) cells. Immunohistochemically detectable HA in PT (panels D-F) was not affected by CD44 silencing. The figure shows images of representative 6 µm paraffin sections with cell nuclei counterstained with hematoxylin. Bars, 100 µm.

**Figure 5:**
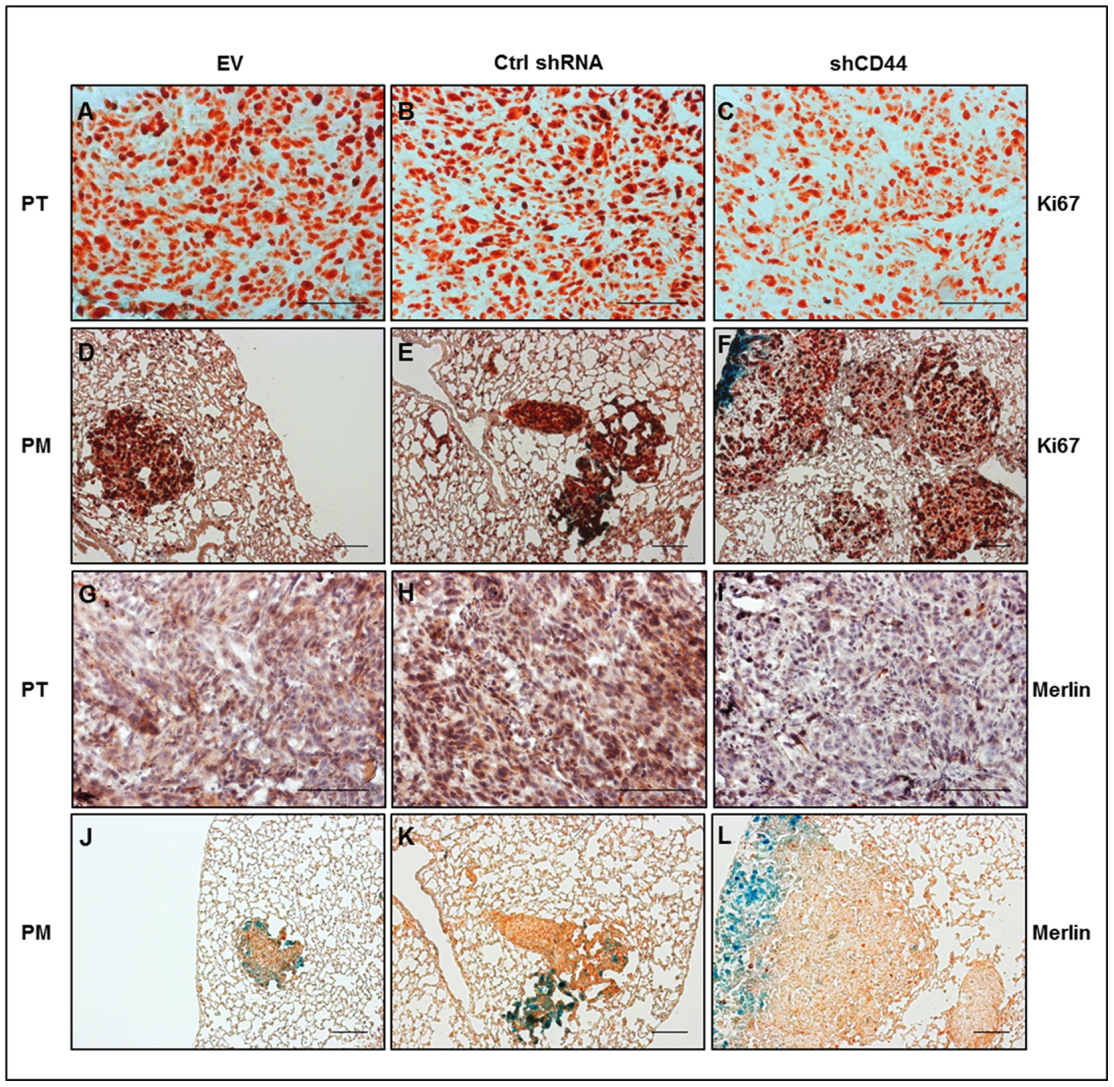
CD44 silencing in 143-B OS cells reduced the density of proliferating cells in intratibial primary tumors and decreased the content of the tumor suppressor merlin in primary tumors and pulmonary metastases to immunohistochemically nearly undetectable levels. Representative images of 6 µm paraffin sections of primary tumor (PT) (panels A-C and G-I) and of lung tissue with pulmonary metastases (PM) (panels D-F and J-L) stained for the proliferation marker Ki67 (panels A-F) and for merlin (panels G-L). Tissues were collected at sacrifice from SCID mice intratibially injected with 143-B EV (EV), 143-B Ctrl shRNA (Ctrl shRNA) or 143-B shCD44 (shCD44) cells. Bars, 100 µm.

In view of the discrepant malignant properties of 143-B shCD44 cells *in vitro* and *in vivo*, we assessed the expression of immunoreactive CD44 by immunohistochemistry with Hermes3 antibodies in primary tumor tissue and lung metastases derived from 143-B shCD44 cells and compared it with 143-B EV and 143-B Ctrl shRNA cell-derived tumors. This analysis demonstrated that CD44 silencing was maintained *in vivo* in 143-B shCD44 cells (Figure 4C and I). The content of HA in the extracellular matrix of primary tumors, on the other hand, was indistinguishable in 143-B shCD44, -EV and -Ctrl shRNA cell-derived tumors, indicating that the levels of expression of CD44 gene products in 143-B cells did not affect extracellular HA deposition (Figure 4D-F). Interestingly, nuclear Ki67 immunostaining of proliferating tumor cells on primary tumor and lung paraffin sections showed a specific structure of primary tumors and lung metastases in mice bearing tumors depleted of CD44. 143-B shCD44 cells appeared to be in much looser contact in primary tumors and metastases than 143-B EV and 143-B Ctrl shRNA cells that formed remarkably denser malignant tissue than 143-B shCD44 cells (Figure 5A-F). The discrepant effects of CD44 silencing in 143-B cells observed *in vitro* and *in vivo* suggested different responses to this manipulation of cells in culture and in the living animal. Merlin, known to have tumor suppressor activity upon binding to the cytoplasmic domain of HA-interacting CD44 and thereby conferring cell growth arrest by contact inhibition [27], was considered as a candidate molecule contributing to the regulation of 143-B cell aggressiveness. Immunohistochemistry on intratibial primary tumor and lung paraffin sections indeed showed remarkably lower merlin protein levels in 143-B shCD44 than in 143-B EV and 143-B Ctrl shRNA cell-derived tumors and pulmonary metastases (Figure 5). Such differences in merlin protein levels in the three manipulated cell lines were not observed *in vitro* (not shown).

## Discussion

Only a few studies addressed so far the biological relevance of CD44 expression in OS progression and metastasis and the results are controversial. One study did not find a correlation between CD44 protein expression and overall survival of OS patients [28] and in a second study, OS patients with high CD44 mRNA expression levels in primary tumor tissue were found to be more prone to have metastases than those with low expression [29]. Moreover, expression of distinctive CD44 isoforms was found to be correlated with unfavorable prognosis [16], [17].

Here, we aimed at determining the role of CD44 in the human 143-B OS cell line with metastatic activity, which has robust endogenous CD44 expression and was selected as representative for OS. Therefore, we silenced the expression of CD44 gene products in 143-B cells and studied its impact *in vitro* and *in vivo* in an intratibial xenograft OS model in SCID mice. Interestingly, we obtained discrepant findings *in vitro* and *in vivo*. *In vitro*, stable shRNA-mediated CD44 knock-down significantly suppressed cell migration and growth in soft agar, implying that CD44 may contribute to the increased metastatic properties of 143-B cells. However, upon intratibial injection into SCID mice, 143-B cells with reduced CD44 expression even enhanced the malignant phenotype when compared to control cells. Mice bearing shCD44 xenografts developed larger primary tumors and had significantly increased number of pulmonary metastases when compared to those in control animals. In contrast to our *in vitro* data, experiments *in vivo* using the orthotopic xenograft mouse model identified CD44 as a metastasis suppressor gene in 143-B cells.

A plausible explanation for the enhanced metastatic activity in cells depleted of CD44 is their decreased adhesion to HA-rich extracellular matrices within primary tumors, consistent with the reduced adhesion to HA that we observed *in vitro*. Furthermore, histology of tumor tissue revealed a low adhesive structure of 143-B shCD44 cells with wide intercellular gaps, as opposed to control transduced cells. The changes in adhesive properties of shCD44 cells may also have facilitated their mobility and enabled expansion and dissemination beyond the primary tumor site, ultimately leading to elevated metastatic potential. A study performed by Lopez et al. is in good agreement with our concept, in which they report that CD44 loss has a metastasis-promoting effect in a mouse model of spontaneously metastasizing breast cancer [30]. Moreover, the authors show that CD44/HA interactions inhibit invasion in a three-dimensional *in vitro* invasion assay, suggesting that CD44 engagement with HA is protective against metastasis. Another explanation for the elevated malignant phenotype in 143-B cells with silenced CD44 is the observed loss of merlin protein expression *in vivo* in these cells. Merlin is encoded by neurofibromatosis type 2 (NF2) gene and mutations and deletions of merlin underlie NF2 familial cancer syndrome, characterized by development of schwannomas, meningiomas and ependymomas [31]. As mutations of NF2 were also detected in other cancer types, it is considered as a tumor suppressor gene in a wide variety of tumor cells. Merlin is a multifunctional protein that regulates cell shape, proliferation, survival, motility and invasion. Interestingly, mice heterozygous for a mutation at the NF2 locus (Nf2+/-) are cancer prone, and develop a wide spectrum of tumors, most frequently OS, that display strikingly high metastatic proclivity, unlike the benign tumors in human patients with NF2 syndrome [32]. This study provided experimental support for the association of NF2 loss and elevated metastatic potential. Conversely to the observations in mice, in human OS patient samples NF2 mutations could not be found, whereas merlin protein could be detected, implicating the apparent differences between the mouse and human OS tumorigenesis [33]. Nevertheless, as merlin functions as a tumor and metastasis suppressor, accelerated tumor growth and enhanced ability to form metastases seen in the intratibial OS mouse model presented here may be the consequence of loss of merlin's expression in 143-B shCD44 cells. In breast cancer, in which mutations are absent as in OS, the stability of merlin mRNA was found unaltered [34]. A recent report by Morrow et al. showed that the loss of merlin protein observed in breast cancer tissues occurs without any change at the transcript level and is a result of proteasomal degradation induced by osteopontin initiated Akt-mediated phosphorylation of merlin [35]. Mechanisms underlying merlin loss in OS cells need yet to be elucidated. Additionally, merlin has been reported to reverse the Ras-induced malignant phenotype [36]. Given the fact that 143-B cells were generated through Ki-Ras transformation [37], we suggest that Ras-driven metastatic behavior is even more pronounced upon loss of merlin protein expression *in vivo*. Therefore, we cannot exclude the possibility of a cell-type specific effect of CD44 down-regulation on tumor and metastasis formation. However, CD44 may act as a metastasis suppressor by regulating merlin expression or function in a subset of OS where Ras signaling is involved. RHAMM, another HA binding protein that is expressed in 143-B cells (not shown), might be an additional player in our model. Nedvetzki et al. showed that RHAMM can compensate for the loss of CD44 in binding HA, thereby supporting migration in a model of collagen-induced arthritis [38]. The observed compensation in the above mentioned model was not due to an increase in RHAMM expression, but rather by enhanced HA-induced signaling through RHAMM. The potential role of RHAMM in our OS model will be subject of a future detailed study.

In conclusion, the findings of our study imply that CD44 is a negative regulator of metastasis in 143-B OS cells. The apparent discrepancy between *in vitro* and *in vivo* outcomes of CD44 knock-down on tumorigenic and metastatic properties of 143-B cells highlights the essential impact of the tumor environment on OS progression. CD44 functions as a metastasis suppressor gene in this particular experimental OS. Although the 143-B cells were representative for the expression pattern of CD44 gene products observed in other cell lines, the here observed effects of CD44 silencing might be particularly important in OS cells with up-regulated Ras activity. Future studies investigating CD44 expression in large cohorts of human tumor tissue samples will further contribute to the delineation of its role in OS.

## Supporting Information

Figure S1 **Cell cycle analysis of 143-B EV, 143-B Ctrl shRNA or 143-B shCD44 cells.** Cell cycle progression was measured by propidium iodide (PI) staining using flow cytometry. Briefly, cells were trypsinized, washed once with cold PBS and resuspended in 300 µl of cold PBS. Subsequently, cells were fixed in ice cold ethanol and stored at −20°C overnight. The next day, DNA was stained in PI/RNase staining buffer (BD Pharmingen AG, Allschwil, Switzerland) at 37°C for 30 min in the dark. The samples were analysed on a FACS machine (Calibur, BD) and the cell cycle distribution was calculated using FlowJo software. The values indicate the mean ± SEM of six analyses from two independent samples.
(TIF)
