# Supplementary figures and images for: Silencing of CD44 Gene Expression in Human 143-B Osteosarcoma Cells Promotes Metastasis of Intratibial Tumors in SCID Mice
Source: PLoS One. 2013 Apr 2;8(4):e60329. doi: 10.1371/journal.pone.0060329 (PMC3614951; doi:10.1371/journal.pone.0060329)

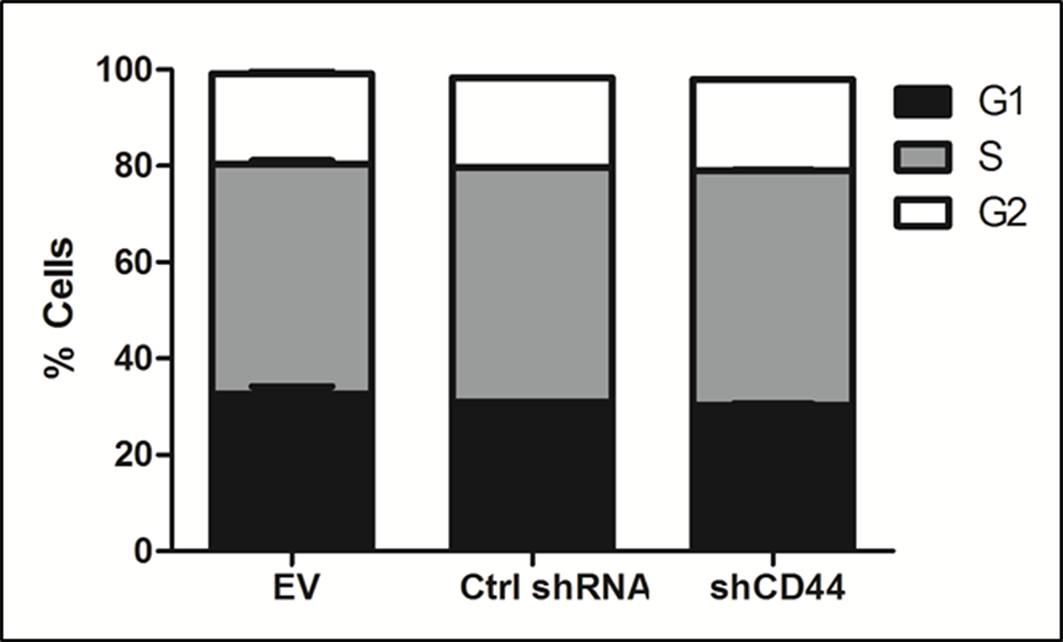

Supplement: Figure S1 — Cell cycle analysis of 143-B EV, 143-B Ctrl shRNA or 143-B shCD44 cells. Cell cycle progression was measured by propidium iodide (PI) staining using flow cytometry. Briefly, cells were trypsinized, washed once with cold PBS and resuspended in 300 µl of cold PBS. Subsequently, cells were fixed in ice cold ethanol and stored at −20°C overnight. The next day, DNA was stained in PI/RNase staining buffer (BD Pharmingen AG, Allschwil, Switzerland) at 37°C for 30 min in the dark. The samples were analysed on a FACS machine (Calibur, BD) and the cell cycle distribution was calculated using FlowJo software. The values indicate the mean ± SEM of six analyses from two independent samples. (TIF) [file pone.0060329.s001.tif]
